# Diagnostic and Prognostic Value of Plasma GFAP in Sporadic Creutzfeldt–Jakob Disease in the Clinical Setting of Rapidly Progressive Dementia

**DOI:** 10.3390/ijms25105106

**Published:** 2024-05-08

**Authors:** Giuseppe Mario Bentivenga, Simone Baiardi, Andrea Mastrangelo, Corrado Zenesini, Angela Mammana, Marcello Rossi, Barbara Polischi, Sabina Capellari, Piero Parchi

**Affiliations:** 1 Department of Biomedical and Neuromotor Sciences (DiBiNeM), University of Bologna, 40139 Bologna, Italy; giuseppe.bentivenga@studio.unibo.it (G.M.B.);; 2 IRCCS Istituto delle Scienze Neurologiche di Bologna, 40139 Bologna, Italy

**Keywords:** prion, Creutzfeldt–Jakob disease, Alzheimer’s disease, GFAP, biomarker, co-pathology, neurodegeneration, neuroinflammation

## Abstract

The diagnostic and prognostic value of plasma glial fibrillary acidic protein (pl-GFAP) in sporadic Creutzfeldt–Jakob disease (sCJD) has never been assessed in the clinical setting of rapidly progressive dementia (RPD). Using commercially available immunoassays, we assayed the plasma levels of GFAP, tau (pl-tau), and neurofilament light chain (pl-NfL) and the CSF total tau (t-tau), 14-3-3, NfL, phospho-tau181 (p-tau), and amyloid-beta isoforms 42 (Aβ_42_) and 40 (Aβ_40_) in sCJD (*n* = 132) and non-prion RPD (np-RPD) (*n* = 94) patients, and healthy controls (HC) (*n* = 54). We also measured the CSF GFAP in 67 sCJD patients. Pl-GFAP was significantly elevated in the sCJD compared to the np-RPD and HC groups and affected by the sCJD subtype. Its diagnostic accuracy (area under the curve (AUC) 0.760) in discriminating sCJD from np-RPD was higher than the plasma and CSF NfL (AUCs of 0.596 and 0.663) but inferior to the 14-3-3, t-tau, and pl-tau (AUCs of 0.875, 0.918, and 0.805). Pl-GFAP showed no association with sCJD survival after adjusting for known prognostic factors. Additionally, pl-GFAP levels were associated with 14-3-3, pl-tau, and pl-NfL but not with CSF GFAP, Aβ_42_/Aβ_40_, and p-tau. The diagnostic and prognostic value of pl-GFAP is inferior to established neurodegeneration biomarkers. Nonetheless, pl-GFAP noninvasively detects neuroinflammation and neurodegeneration in sCJD, warranting potential applications in disease monitoring.

## 1. Introduction

Sporadic Creutzfeldt–Jakob disease (sCJD) is the most common humanprion disease, a rare group of neurodegenerative disorders related to prion protein (PrP) misfolding. It encompasses six major clinicopathological subtypes that are primarily determined by the genotype at the polymorphic codon 129 (encoding methionine, M, or valine, V) of the prion protein gene (*PRNP*) and the type (1 or 2) of misfolded PrP (PrP^Sc^) accumulating in the brain (e.g., MM1, MV1 (together known as MM(V)1), VV1, MM2, etc.). Each subtype presents distinctive pathological features and regional distribution patterns. For instance, while MM(V)1 patients show significant involvement (in terms of both neuronal loss and gliosis) of the neocortex (especially in the occipital lobe), striatum, thalamus, and cerebellum, VV2 patients present a prominent subcortical and cerebellar pathology, with only late cortical involvement (the occipital lobe is the least affected). Different sCJD subtypes also exhibit distinct clinical characteristics. For example, the most common sCJD subtypes (MM(V)1 and VV2) are characterised by short survival (on average, 4 and 6.5 months, respectively), while “atypical” and rarer subtypes (MV2K, MM2C, MM2T, and VV1) may exhibit a long disease duration, usually lasting more than one year [1,2].

Although sCJD is a common cause of rapidly progressive dementia (RPD), its early diagnosis and prognostication remain challenging due to heterogeneous clinical phenotypes and disease course. In recent years, the introduction of cerebrospinal fluid (CSF) biomarkers of neurodegeneration and prion pathology (i.e., the prion real-time quaking-induced conversion (RT-QuIC) assay) and brain magnetic resonance imaging (MRI) have dramatically improved the in vivo identification of sCJD patients and demonstrated some prognostic potential [3,4,5,6,7,8,9,10,11,12]. However, given the invasiveness of lumbar puncture (LP) and the specialised expertise required by these techniques (e.g., RT-QuIC or MRI), the identification of blood biomarkers for screening,, prognostication and disease monitoring remains a research priority [13].

Based on the notion that microgliosis and astrogliosis are core pathological events in sCJD and that astrocytes are increasingly recognised as contributors to PrP^Sc^ propagation and cell death, likely influencing disease progression, current research is increasingly focusing on “neuroinflammatory” biomarkers [14,15,16,17]. In this regard, glial fibrillary acidic protein (GFAP), a well-known marker of astrogliosis, has been reported to increase significantly in the CSF of sCJD patients compared to healthy controls and other neurodegenerative disorders [15]. The development of novel assays, such as single-molecule arrays (SiMOA), has enabled the detection of numerous proteins, including GFAP, in the blood with high accuracy. However, studies regarding the diagnostic performance of plasma GFAP (pl-GFAP) in the real-life clinical setting of an RPD cohort are currently lacking. We measured pl-GFAP levels in a large RPD cohort comprising sCJD, non-prion RPD (np-RPD) patients, and healthy controls (HCs). We compared their diagnostic accuracy to the one provided by other traditional CSF and blood surrogate neurodegeneration biomarkers. We also studied the distribution of pl-GFAP levels across different sCJD clinicopathological subtypes and evaluated their association with clinical variables, such as disease stage and survival in sCJD.

## 2. Results

### 2.1. Demographic Variables and CSF Biomarkers Value Distribution in the Diagnostic Groups

Demographic variables and CSF biomarkers’ results in the main diagnostic groups are reported in Table 1.

Age was associated with the pl-GFAP levels in the sCJD (rho = 0.318, *p* = 0.0002) and np-RPD (rho = 0.258, *p* = 0.0118) groups, with plasma neurofilament light chain (pl-NfL) in the sCJD (rho = 0.313, *p* = 0.0003) group, with phospho-tau181 (p-tau) in the sCJD (rho = 0.224, *p* = 0.0157) and np-RPD (rho = 0.284, *p* = 0.0072) groups, and with the amyloid beta-42/amyloid beta-40 (Aβ_42_/Aβ_40_) ratio in the sCJD (rho = 0.306, *p* = 0.0009) and np-RPD (rho = 0.339, *p* = 0.0013) groups. In contrast, age did not show any association with the biomarker values in the HC. Accordingly, we adjusted for age all analyses, including pl-GFAP or pl-NfL and p-tau or the Aβ_42_/Aβ_40_ ratio. Sex showed no effect on the plasma and CSF biomarker values. There were no significant differences in sex distribution among the three diagnostic groups. Conversely, age differed between the np-RPD patients and both the sCJD (*p* = 0.0003) and HC (*p* < 0.0001) groups, with the former being older than the latter two (*p* = 0.0006).

**Figure 1:**
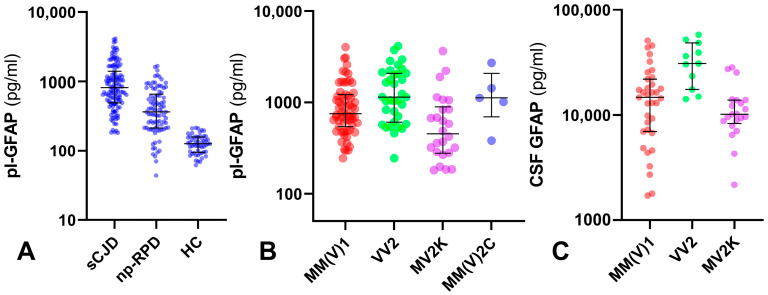
Biomarker levels in the main diagnostic groups (sCJD, np-RPD, and HC) and sCJD subtypes (**A**). Distribution of the GFAP values in the main sCJD subtypes in plasma (**B**) and CSF (**C**). Thick lines represent medians and interquartile ranges. Plasma and CSF GFAP values are expressed in a logarithmic scale. See the main text for all the *p*-values (Kruskal–Wallis, followed by Dunn–Bonferroni post hoc test). Only sCJD subgroups comprising at least three cases are shown. Abbreviations: CSF, cerebrospinal fluid; GFAP, glial fibrillary acidic protein; HC, healthy control; np-RPD, non-prion rapidly progressive dementia; pl-, plasma; sCJD, sporadic Creutzfeldt–Jakob disease.

Prion patients showed higher levels of pl-GFAP in comparison to the HC (*p* < 0.0001) and np-RPD participants (*p* < 0.0001). Moreover, pl-GFAP levels were higher in the np-RPD group than in the HCs (*p* < 0.0001). All analyses remained significant after adjusting for age at sampling (*p* < 0.0001 for all comparisons) (Figure 1).

### 2.2. Correlations between Plasma and CSF GFAP and Other Biomarkers

In the sCJD cohort, the pl-GFAP levels significantly correlated with the pl-NfL (rho = 0.502, *p* < 0.0001), plasma tau (pl-tau) (rho = 0.253, *p* = 0.0058), 14-3-3 (rho = 0.370, *p* < 0.0001), total tau (t-tau) (rho = 0.329, *p* < 0.0001), and the Aβ_42_/Aβ_40_ ratio (rho = −0.204, *p* = 0.0285), with the two latter correlations losing significance after adjusting for age. Similarly, the CSF GFAP values were significantly associated with the 14-3-3 (rho = 0.386, *p* < 0.0014), t-tau (rho = 0.439, *p* = 0.0002), and p-tau (rho = 0.469, *p* < 0.0001), but not with the CSF NfL, pl-NfL, pl-tau, or the Aβ_42_/Aβ_40_ ratio. CSF GFAP was not significantly associated with pl-GFAP in the whole sCJD cohort or within the main clinicopathological subtypes (i.e., MM(V)1, VV2, MV2K). In the np-RPD cohort, pl-GFAP significantly correlated with p-tau (rho = 0.264, *p* = 0.0129), the Aβ_42_/Aβ_40_ ratio (rho = −0.338, *p* = 0.0014), pl-NfL (rho = 0.330, *p* = 0.0013), pl-tau (rho = 0.210, *p* = 0.0487), and t-tau (rho = 0.211, *p* = 0.0405), with the three latter associations losing significance after adjusting for age at sampling.

### 2.3. Distribution of Plasma and CSF GFAP Levels in the sCJD Cohort According to Clinicopathological Subtypes

After stratification according to the sCJD subtype and considering the age adjustment, MM(V)1, VV2, and MM2C patients showed significantly higher levels of pl-GFAP compared to those with MV2K (MM(V)1 vs. MV2K, *p* = 0.004; VV2 vs. MV2K, *p* < 0.001; MM(V)2C vs. MV2K, *p* = 0.017). VV2 participants had higher pl-GFAP values than the MM(V)1 group (*p* = 0.035). All findings previously mentioned remained statistically significant after excluding the probable sCJD patients. Similarly, the CSF GFAP levels were higher in VV2 compared to MM(V)1 (*p* = 0.001) and MV2K (*p* < 0.001), even after excluding the probable sCJD participants. The MM(V)1 group showed tendentially higher values than the MV2K, although this result missed the significance threshold. Plasma and CSF GFAP level distribution among CJD subtypes is shown in Table 2 and Figure 1.

The profiles of the remaining plasma and CSF surrogate biomarkers of neurodegeneration stratified by prion disease subtypes are shown in Table S1 [18].

### 2.4. Distribution of Plasma GFAP Levels in the sCJD Cohort According to A/T Status

Next, to test whether pl-GFAP levels in sCJD were significantly influenced by amyloid and tau copathologies, we stratified patients according to their A/T status. The A+ sCJD group showed higher pl-GFAP levels than A- cases (*p* = 0.0028); however, this result was no longer significant after age adjustment. Pl-GFAP was higher in T+ sCJD vs. T- sCJD patients (*p* = 0.0026), even after accounting for age (*p* = 0.024); however, there were no significant differences when stratifying for clinicopathological subtypes (MM(V)1 and VV2).

### 2.5. Diagnostic Performance of Plasma GFAP in the Differential Diagnosis between sCJD and np-RPD

**Figure 2:**
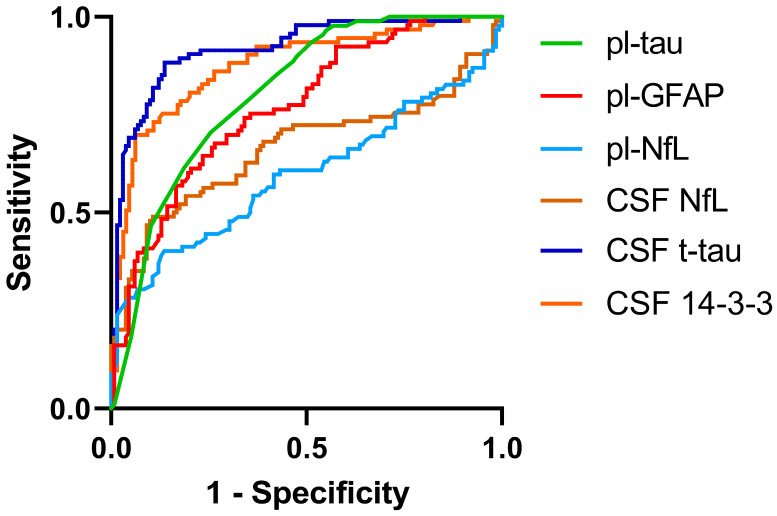
Biomarker diagnostic performance. ROC curves for plasma GFAP (red), plasma tau (green), plasma NfL (light blue), CSF NfL (brown), CSF t-tau (blue), and CSF 14-3-3 (orange) in their comparisons between the CJD and np-RPD groups. Abbreviations: CJD, Creutzfeldt–Jakob disease; CSF, cerebrospinal fluid; GFAP, glial fibrillary acidic protein; NfL, neurofilament light chain; np-RPD, non-prion rapidly progressive dementia; pl-, plasma; ROC, receiver operating characteristic; t-tau, total tau.

To assess the diagnostic performance of CSF and plasma biomarkers, we calculated the receiver operating characteristic curves (ROCs), sensitivity, and specificity for all biomarkers. Detailed ROC curve analyses for the CSF biomarkers are reported in Table 3 and Figure 2.

In the ROC curve analysis, pl-GFAP yielded a diagnostic accuracy of 76% (area under the curve (AUC) of 0.760 (0.697–0.823)) in discriminating between CJD and np-RPD. Its diagnostic performance was in the range of pl-tau (AUC of 0.805 (0.746–0.863)) and exceeded that of pl-NfL (AUC of 0.596 (0.515–0.677)) (pl-GFAP vs. pl-NfL: *p* = 0.0454), and CSF NfL (AUC of 0.663 (0.583–0.742)) (CSF NfL vs. pl-GFAP: *p* = 0.0152); however, it was lower than that of 14-3-3 (AUC of 0.875 (0.827–0.924)) (14-3-3 vs. pl-GFAP: *p* = 0.0019), and t-tau (AUC if 0.918 (0.880–0.956)) (t-tau vs. pl-GFAP: *p* < 0.0001).

When analysing the biomarkers’ diagnostic accuracy in discriminating between patients with sCJD and those with a neurodegenerative RPD (rp-ND), pl-GFAP (AUC of 0.762 (0.688–0.837)) was again outperformed by 14-3-3 and t-tau (pl-GFAP vs. 14-3-3, *p* < 0.0001; pl-GFAP vs t-tau, *p* < 0.0001), while its diagnostic performance was in the range of CSF NfL, pl-NfL and pl-tau. Conversely, when we restricted the analysis to the non-neurodegenerative RPD (rp-nonND) group, pl-GFAP (AUC of 0.758 (0.673–0.843)) diagnostic accuracy was in the range of pl-tau and higher than both CSF and plasma NfL (pl-GFAP vs. CSF NfL: *p* = 0.0004; pl-GFAP vs. pl-NfL: *p* < 0.0001), although it was still outperformed by 14-3-3 and t-tau (pl-GFAP vs. 14-3-3, *p* < 0.0001; pl-GFAP vs. t-tau, *p* < 0.0001).

In the differential diagnosis between atypical, slowly progressive sCJD individuals (i.e., MV2K, MM(V)2C, MM2T, and VV1) and np-RPD patients, pl-GFAP (AUC of 0.614 (0.500–0.728)) diagnostic performance was in the range of that of 14-3-3, pl-tau, and CSF and plasma NfL, and inferior to that of t-tau (pl-GFAP vs. t-tau, *p* = 0.0177).

### 2.6. Prognostic Value and Distribution according to Disease Stages of Plasma GFAP in sCJD

Next, we used univariable and multivariable Cox regression analyses to investigate the associations among survival, biomarkers’ values, and other variables known as prognostic factors in prion disease (age at sampling, time from symptoms onset to sample collection, and codon 129 genotype). We evaluated the association of survival with both the continuous values and tertiles of each biomarker, i.e., we investigated how survival changed in subjects with higher biomarker levels (belonging to the “mid-tertile” and “high-tertile” groups) compared to those with lower biomarker levels (“low-tertile” group).

**Figure 3:**
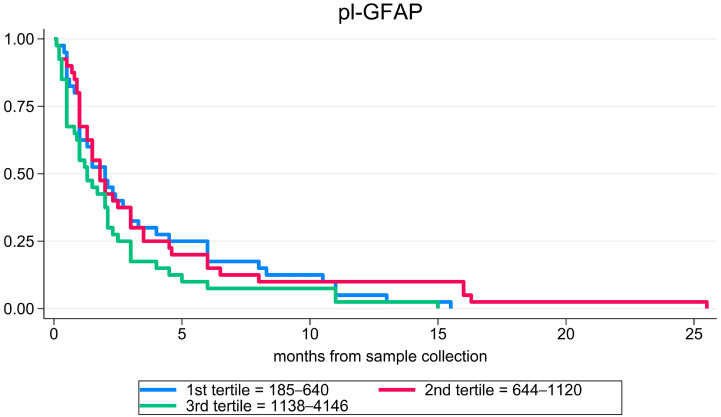
Plasma GFAP prognostic performance. Survival curves in patients of the whole sCJD cohort according to the values of plasma GFAP. Abbreviations: pl-GFAP, plasma glial fibrillary acidic protein.

When considering the whole sCJD cohort, pl-GFAP was significantly associated with survival (hazard ratio (HR) 1.27 (1.00–1.63), *p* = 0.050); however, this result lost significance after accounting for known prognostic factors in prion disease. Moreover, we found no significant associations between pl-GFAP and survival when stratifying for the clinicopathological subtype, neither in the univariate nor multivariate Cox regression. Detailed data regarding the association between pl-GFAP levels and survival are shown in Table 4. Survival curves are shown in Figure 3.

Survival analyses for the remaining biomarkers are reported in Table S2 and shown in Figure S1.

Regarding the possible correlation between plasma and CSF GFAP and disease stage within the whole sCJD cohort, higher pl-GFAP was weakly associated with a later disease stage (rho = 0.263, *p* = 0.0035), even after age adjustment (*p* = 0.016), while CSF GFAP showed no significant association, even after accounting for age. When stratifying for sCJD subtypes, the disease stage did not correlate with the plasma or CSF GFAP values.

## 3. Discussion

Neuroinflammation is increasingly recognised as a pivotal pathogenetic process in neurodegenerative disorders, including prion disease [14,15,16]. In this regard, proteins released in body fluids due to the neuroinflammatory response may represent promising biomarkers for diagnosis and disease monitoring. While the diagnostic potential of CSF GFAP has been previously explored in prion disease [15], little is known about its diagnostic value in blood. Moreover, its relative prognostic value compared to the currently available blood biomarkers (e.g., pl-NfL or pl-tau) is still mostly unexplored. In this study, we measured pl-GFAP in a large RPD cohort comprising both sCJD and np-RPD patients and healthy subjects. We reported a marked increase in the pl-GFAP levels of sCJD patients compared to the np-RPD patients and HCs, likely reflecting the significant astrocyte activation occurring in prion disease as a response to PrP^Sc^ misfolding and aggregation [16,17].

When evaluating its diagnostic accuracy in distinguishing sCJD from np-RPD individuals, pl-GFAP performed similarly to pl-tau (to date, one of the most accurate biomarkers available in plasma [18,19,20]) and better than plasma and CSF NfL, although significantly worse than traditional CSF biomarkers (14-3-3, t-tau). This result was substantially consistent even after stratifying the np-RPD in the rp-ND and rp-nonND subgroups, and also when considering only rp-AD participants (i.e., the most common form of rp-ND) (data not shown). Interestingly, the diagnostic performance of pl-GFAP was also substantially maintained for the atypical sCJD subtypes (MV2K, MM2C, MM2T, and VV1), which often show low levels of traditional plasma and CSF biomarkers, yielding an accuracy against np-RPD in the ranges of CSF 14-3-3, CSF and plasma NfL, and pl-tau. In summary, despite the fair diagnostic performance, our results indicate that pl-GFAP has no added value compared to traditional CSF and plasma surrogate markers of neurodegeneration. Moreover, the recent discovery of blood markers with comparable accuracy to CSF 14-3-3 and t-tau (e.g., β-synuclein) further reduces the potential applications and usefulness of pl-GFAP for diagnostic/screening purposes [21].

We also evaluated the prognostic value of pl-GFAP compared to other biomarkers. The pl-GFAP levels were significantly associated with survival in the whole sCJD cohort. However, this result missed the significance threshold after accounting for covariates known to have a prognostic role in prion disease (age at sampling, *PRNP* genotype at codon 129, and time between symptoms onset and sampling) and after stratifying the analysis for sCJD subtypes. This result confirms, in a larger group of patients, the modest prognostic value of pl-GFAP, previously reported in a small CJD cohort [12]. Similarly, the CSF GFAP levels were not significantly associated with survival either in the univariable or in the multivariable analyses. Conversely, all CSF and plasma surrogate neurodegeneration biomarkers could accurately predict survival, as previously reported [12,18]. The reasons for the poor prognostic value of GFAP compared to neurodegeneration markers in sCJD are not known. Intriguingly, in other neurodegenerative diseases (e.g., Alzheimer’s disease (AD), GRN-frontotemporal dementia (GRN-FTD)), and even in older people at high risk for dementia, elevated blood GFAP levels have been reported to have some predictive value, being associated with faster cognitive decline and reduced brain volumes [22]. These differences in the pl-GFAP prognostic values among heterogeneous diseases might be due to the different roles that processes of neurodegeneration and astrocytic activation have in determining clinical progression. We speculate that while in sCJD, early massive neurodegeneration occurs, driving clinical deterioration, followed by less timely astrocytic activation (especially in some subtypes, such as VV2) [17], in other more slowly progressive diseases (e.g., AD), neurodegeneration and astrocyte activation may be more temporally linked, and thus, both significantly related to clinical course. Future studies should investigate the heterogeneity of the astrocytic activation process among neurodegenerative diseases and its clinical correlates.

Regarding the association between biomarker levels and disease stage in the CJD cohort, we found a gradual increase in pl-GFAP levels along the disease course. However, considering each clinicopathological subtype separately, the disease stage did not correlate with pl-GFAP concentrations. CSF GFAP showed a similar trend, as we found no association between its CSF concentrations and disease stage in the CJD cohort or each clinicopathological subtype. Overall, this evidence suggests that plasma and CSF GFAP levels increase in the early symptomatic phase and remain substantially stable during the disease course. Future studies, including asymptomatic individuals at risk of prion disease, should help investigate GFAP dynamics in the presymptomatic disease stage.

Regarding the pl-GFAP distribution along the sCJD spectrum, VV2 patients showed higher plasma levels than MM(V)1, and both had higher concentrations than MV2K. Similarly, CSF GFAP concentrations were higher in VV2 than in both MM(V)1 (with an even higher difference than in the plasma) and MV2K, as previously reported [15]. The presence of two slightly distinct biofluids GFAP profiles (with a lowering of biomarker levels in VV2 relative to MM(V)1 in plasma compared to CSF) justifies, at least in part, the poor correlation between the CSF and pl-GFAP levels. This peculiar behaviour, already observed with other plasma biomarkers, such as tau, NfL, and β-synuclein, could be related to the different regional lesion profiles between the two sCJD subtypes. Specifically, the early cortical involvement (also in terms of astrogliosis), which characterises MM(V)1 (but not VV2) subjects, may lead to a higher spillover of these molecules in the blood compared to that from subcortical regions [2,17,18,21].

The reasons for different levels of CSF GFAP among the main sCJD subtypes (i.e., MM(V)1, VV2, and MV2K) are unknown. A previous study reported that the brains of the three major sCJD subtypes do not present significant differences in total GFAP immunoreactivity [17]. In this regard, the higher CSF concentrations observed in VV2 compared to MM(V)1 and MV2K may reflect reduced CSF GFAP drainage, possibly due to lower cortical astrogliosis (and thus, GFAP drainage in the blood) in the former compared to the other subtypes, in line with our previous hypothesis [17].

This study also studied the possible inter-correlation between GFAP and markers indicative of alternative pathological processes. In the sCJD cohort, both plasma and CSF GFAP variably correlated with plasma and CSF neurodegeneration biomarkers, likely reflecting the close relationship between neuroinflammation and neurodegeneration, as previously described [16,17], but not with AD core biomarkers (p-tau and the Aβ_42_/Aβ_40_ ratio). In contrast, in the np-RPD cohort, the pl-GFAP levels were also significantly associated with AD core biomarkers and neurodegeneration markers, most likely due to the inclusion of many AD patients in the np-RPD group. Pl-GFAP levels are indeed markedly increased in AD compared to healthy controls or other neurodegenerative diseases (e.g., FTD). In subjects with AD, pl-GFAP levels strongly correlate with cortical Aβ deposition. More specifically, linear, positive associations were observed in the early stages and diverged during the disease course, suggesting that astrocytic activation begins in the presymptomatic phase of AD and is associated with brain Aβ load [22]. The nonspecific significance of increased pl-GFAP levels makes it an unreliable marker in the differential diagnosis between sCJD and AD. In this clinical setting, the diagnostic accuracy of proposed AD plasma biomarkers, which are expected to specifically reflect AD core neuropathological changes (e.g., different phosphorylated tau isoforms), should be addressed in future studies.

Next, we used our wide and heterogeneous sCJD cohort to test whether pl-GFAP levels in sCJD were significantly influenced by amyloid (A+) or tau (T+) copathologies. The pl-GFAP levels were not significantly different between the A+ and A- sCJD patients. Conversely, the T+ sCJD participants showed higher pl-GFAP than the T- ones; however, this result was not significant after stratifying for the clinicopathological subtype. This apparently contradictory result is most likely due to the inclusion, when considering the whole sCJD cohort, with many sCJD patients showing both high pl-GFAP and CSF p-tau concentrations. Indeed, VV2 patients often exhibit high p-tau levels (and thus, frequent T+ status), reflecting either an AD tauopathy or a prion disease-related tauopathy, or both [23]. This evidence suggests that neither A+ nor T+ status significantly influences pl-GFAP concentrations in sCJD.

This study comes with some limitations. First, it cannot be ruled out that some patients without neuropathological evaluation were misdiagnosed. Moreover, classifying probable sCJD patients into a specific clinicopathological subtype could have been inaccurate. However, we believe that the use of second-generation prion RT-QuIC and codon 129 genotyping have effectively minimised these risks. We also recognise that small sample sizes, especially in analyses involving atypical sCJD subtypes, and the lack of clinical measurements of functional status and progression rate are further limitations. As an additional limitation, the fact we used akinetic mutism in place of time to death when life-extending treatments were adopted might have introduced a bias in the survival calculation, given that we did not use the same variable for all patients. Ultimately, the validity and generalizability of our results are limited by the retrospective and unicentric design of the study.

## 4. Materials and Methods

### 4.1. Patients’ Selection

We retrospectively analysed CSF and plasma samples from RPD patients submitted from 2003 to 2022 to the Neuropathology Laboratory (NP-Lab) at the Institute of Neurological Sciences of Bologna with suspicion of CJD at the time of the LP for diagnostic purposes. We included patients with a definite (i.e., neuropathological) or probable clinical diagnosis and enough CSF to perform the biomarker assays. The total cohort comprised 226 patients, 132 suffering from sCJD and 94 from np-RPD. We assayed the plasma concentrations of pl-GFAP, pl-NfL, and pl-tau and of the CSF 14-3-3, t-tau, NfL, p-tau, Aβ_42_, and Aβ_40_. The CSF GFAP levels were assessed only in a subgroup of 67 sCJD patients representative of the main clinicopathological subtypes (i.e., MM(V)1, VV2, and MV2K) to study the biomarkers of CSF-plasma dynamics across the most common sCJD subtypes. We also assessed the pl-GFAP concentrations in 54 samples from subjects without evidence of neurological disease (HC) as controls. Specifically, the HCs included a group of healthy (i.e., medical history not relevant for significant diseases/medications) blood donors. Before blood collection, all HCs underwent medical evaluation, including a standardised interview to exclude neurological symptoms and a thorough neurological examination.

Of the 132 sCJD patients, 69 had a neuropathological diagnosis, while 63 had a clinical diagnosis of probable sCJD, according to the current diagnostic criteria [8], and were all positive by prion RT-QuIC. sCJD individuals with a neuropathological diagnosis were also classified into subtypes according to Parchi et al. [2,24] (i.e., MM[V]1, VV2, MV2K, etc.). Among them, 14 participants showing a mixed subtype were classified based on the dominant histotype according to the current criteria [25]. For the biomarker analysis according to the clinicopathological subtype, the patients with definite sCJD were merged with those with a probable diagnosis and a high level of certainty for a given subtype, as previously reported [18,26,27]. Further details regarding the classification of patients with probable sCJD are reported in the Supplementary Materials.

Regarding the biomarkers’ prognostic performance in sCJD, we calculated survival as the time (in months) from sample collection to death or akinetic mutism. The latter was used in place of time to death exclusively when the revision of medical charts indicated the adoption of life-extending treatments (e.g., enteral/parenteral nutrition, tracheostomy). A total of 11 patients were excluded from the survival analyses due to insufficient information on disease duration. Furthermore, the disease stage in the CJD subjects was calculated as the ratio between disease onset to sampling and the overall survival, as reported previously [11,18].

All np-RPD patients presented with RPD and tested negative by prion RT-QuIC. Within the np-RPD cohort, we identified two subgroups depending on the RPD etiology, either degenerative (rp-ND) or not (i.e., RPD due to alternative non-neurodegenerative causes, i.e., rp-nonND]), including inflammatory (e.g., immune-mediated or infectious encephalitis), toxic-metabolic (e.g., Wernicke–Korsakoff syndrome, hepatic encephalopathy, uremia), neoplastic (primitive or secondary CNS malignancies), and vascular (e.g., recurrent or progressive strokes, vascular dementia) etiologies [28,29,30,31,32]. Overall, 15 subjects were diagnosed at autopsy. For the remaining 79 patients, attribution of a high probable clinical diagnosis (i.e., to a particular diagnostic group) was achieved by interpreting clinical, laboratory (e.g., positivity for autoantibodies targeting CNS antigens, positive α-synuclein RT-QuIC assay, and CSF biomarker profile suggestive of AD), and imaging data in light of the most recent RPD diagnostic algorithms [28]. Rp-ND patients with a probable clinical diagnosis were classified according to the current diagnostic criteria and pathology biomarker results (i.e., Aβ_42_/Aβ_40_ ratio, p-tau, and α-synuclein RT-QuIC) [29,30,31,32]. Details regarding the etiologies of all np-RPD participants are presented in Table 5.

### 4.2. CSF Biomarker Analysis

CSF samples were obtained by LP at the L3/L4 or L4/L5 intervertebral level, centrifuged in case of blood contamination, divided into aliquots, and stored in polypropylene tubes at −80 °C until analysis. For the AD core biomarkers measurements, t-tau, p-tau, Aβ_42_, and Aβ_40_ were measured by automated chemiluminescent enzyme immunoassay on the Lumipulse G600II platform (Fujirebio, Gent, Belgium). The inter-assay coefficients of variation (CVs) were <8% for all biomarkers. Pathological values for defining the A/T status were determined using validated cutoff values [33]. More specifically, an Aβ_42_/Aβ_40_ ratio < 0.65 and a p-tau > 62 pg/mL supported the A+ and T+ statuses, respectively.

Commercially available ELISA kits were used to measure the NfL and 14-3-3 gamma isoform, as described [34,35]. The GFAP concentrations were determined by running the commercially available GFAP Discovery Kit (Quanterix) on the SiMOA SR-X platform (Quanterix, Billerica, MA, USA). The intra-assay and the inter-assay CVs were respectively 7% and 15% for NfL, 6% and 13% for 14-3-3, and 8% for GFAP (only one plate was used). Eventually, all CSF samples from patients without autopsy examination, classified as probable sCJD or np-RPD, were tested by the second-generation prion RT-QuIC, as described [6].

### 4.3. Plasma Biomarker Analysis

EDTA plasma samples were collected, aliquoted, and stored at −80 °C according to standard procedures [33]. The pl-NfL, pl-tau, and pl-GFAP were measured with the SiMOA NF-light advantage, SiMOA Human t-tau, and SiMOA GFAP Discovery Kits (i.e., the same used for CSF GFAP quantification) on the SiMOA SR-X platform (Quanterix). The mean intra-assay and inter-assay CVs were 4% and 12% for pl-NfL, 5% and 10% for pl-tau, and 5% and 11% for pl-GFAP.

### 4.4. Statistical Analysis

Statistical analyses were performed using GraphPad Prism 9 (Graph-Pad Software) and Stata 18 SE (StataCorp). The data were expressed as the mean ± standard deviation (SD) or median and interquartile range (IQR) based on the distribution of values. For continuous variables, we variably applied the Mann–Whitney U test, *t*-test, Kruskal–Wallis test (followed by Dunn–Bonferroni post hoc test), or the one-way analysis of variance (followed by Tukey’s post hoc test), depending on the group number and data distribution. All reported *p*-values were adjusted for multiple comparisons, and differences were considered statistically significant at *p* < 0.05. The Chi-square test was adopted for categorical variables. Spearman’s Rho coefficient was used to test the possible correlation among variables. Multivariable linear regression models were used to age-adjust the differences in the biomarker levels among the groups after the transformation of the dependent variable in the natural logarithmic scale. ROC analyses were performed to calculate each biomarker’s sensitivity, specificity, and diagnostic accuracy with a relative 95% confidence interval (95% CI). Maximized Youden’s index was used to define the optimal cutoff value for each biomarker. The areas under the curve between the ROC curves were compared using the DeLong test. The differences were considered statistically significant at a *p*-value < 0.05. For survival analysis, biomarker concentration was naturally log-transformed to fulfil the normal distribution. We used the Kaplan–Meier estimate to calculate the cumulative time-dependent probability of death. Univariable and multivariable Cox regression analyses were performed to assess the association between survival, continuous values or tertiles of each biomarker, and other variables, known as prognostic factors in prion disease (age at sampling, sex, time from symptom onset to sample collection, codon 129 genotype, and clinicopathological subtype) [12,18]. Survival analyses were performed for the whole CJD cohort and in two separate subgroups, according to the clinicopathological subtype, as follows: (1) the most common and rapidly progressive subtypes (i.e., MM(V)1 and VV2), and (2) “atypical CJD”, including all the other subtypes. The survival analysis results are presented as the HRs and 95% CIs. The assumption of proportional hazard was assessed by Schoenfeld residuals.

## 5. Conclusions

In conclusion, our results suggest that pl-GFAP does not provide added diagnostic value compared to the established CSF (14-3-3 and t-tau) and plasma (pl-tau) surrogate biomarkers, yielding a diagnostic performance in the CSF and plasma NfL range. Moreover, our study suggests that pl-GFAP has a worse prognostic potential than all the other CSF and plasma surrogate neurodegeneration biomarkers. Finally, since plasma levels correlate with neurodegeneration markers and are not significantly influenced by amyloid or tau copathology, pl-GFAP may be potentially used to monitor both neuroinflammation and neurodegeneration noninvasively when disease-modifying therapies for sCJD will become available.

## Figures and Tables

**Table 1:** Demographic variables, blood, and CSF biomarkers in the diagnostic groups.

	sCJD (*n* = 132)	np-RPD (*n* = 94)	HC (*n* = 54)	*p* Value
Age at sampling ^1^ (years)	67.9 ± 9.7	73.0 ± 10.9	62.2 ± 4.9	<0.0001
F, *n* (%)	71 (53.8)	47 (50)	20 (37)	0.1100
pl-GFAP ^1^ (pg/mL)	815 (492–1370)	366 (212–684)	126 (95–157)	<0.0001
pl-NfL ^1^ (pg/mL)	116 (63–206)	79 (32–192)	-	<0.0001
pl-tau ^1^ (pg/mL)	9 (4–24)	3 (2–5)	-	<0.0001
CSF 14-3-3 ^1^ (AU/mL)	67,900 (30,200–132,500)	10,900 (6491–22,250)	-	<0.0001
CSF t-tau ^1^ (pg/mL)	6520 (2512–11,575)	610 (405–1327)	-	<0.0001
CSF NfL ^1^ (pg/mL)	7500 (3947–12,300)	2968 (1229–12,313)	-	<0.0001
CSF GFAP ^1,2^ (pg/mL)	14,039 (8787–25,320)	-	-	-
CSF p-tau ^1^ (pg/mL)	61 (38–86)	62 (34–89)	-	0.9564
CSF Aβ_42_/Aβ_40_ ratio ^1^	0.82 (0.72–0.95)	0.62 (0.44–0.90)	-	0.0175

^1^ The age at sampling is expressed as the mean (SD), while the biomarker data are presented as the median (IQR). ^2^ CSF GFAP levels were assayed in a subgroup of 67 sCJD patients. Abbreviations: Aβ, amyloid beta; CSF, cerebrospinal fluid; GFAP, glial fibrillary acidic protein; HC, healthy controls; NfL, neurofilament light chain; np-RPD, non-prion rapidly progressive dementia; pl-, plasma; p-tau, phospho-tau181; sCJD, sporadic Creutzfeldt–Jakob disease; t-tau, total tau.

**Table 2:** Distribution of plasma and CSF GFAP levels in the main subgroups.

Diagnostic Group	N	pl-GFAP (pg/mL)	N	CSF GFAP (pg/mL)
MM(V)1 ^1^	63	753 (541–1227)	35	14,691 (6964–21,955)
VV2 ^1^	35	1143 (609–2080)	11	30,935 (17,516–48,452)
MV2K ^1^	26	454 (281–853)	21	10,164 (8787–13,805)
MM(V)2C ^1^	5	1120 (1013–1438)	-	-
MM2T ^1^	1	701	-	-
VV1 ^1^	2	430, 193	-	-

^1^ Both patients with a definite diagnosis of a specific subtype and patients with a probable diagnosis and a high level of certainty for a given subtype are included. Biomarker data are presented as the median (IQR). Abbreviations: CSF, cerebrospinal fluid; GFAP, glial fibrillary acidic protein; np-RPD, non-prion rapidly progressive dementia; pl-, plasma; sCJD, sporadic Creutzfeldt–Jakob disease.

**Table 3:** Diagnostic performance of plasma GFAP and other surrogate biomarkers.

	**CJD vs. np-RPD**				**Atypical CJD ^1^ vs. np-RPD**			
	**AUC ** **(95% CI)**	**Sens. (%)**	**Spec. (%)**	**Cutoff (pg/mL)**	**AUC** **(95% CI)**	**Sens. (%)**	**Spec. (%)**	**Cutoff (pg/mL)**
CSF t-tau	0.918 (0.880–0.956)	86.2	88.2	1757	0.781 (0.693–0.868)	88.2	64.8	782
CSF 14-3-3	0.875 (0.827–0.924)	93.7	69.8	16,500	0.700 (0.600–0.799)	75.7	69.8	16,500
CSF NfL	0.663 (0.583–0.742)	87.7	20.2	20,500	0.599 (0.499–0.698)	97.0	20.2	20,500
pl-GFAP	0.760 (0.697–0.823)	73.4	67.0	521	0.614 (0.500–0.728)	29.4	91.4	968
pl-tau	0.805 (0.746–0.863)	74.5	70.4	4	0.702 (0.585–0.819)	37.5	97.5	12
pl-NfL	0.596 (0.515–0.677)	95.4	13.0	595	0.514 (0.411–0.617)	100	14.1	404
	**CJD vs. rp-ND**				**CJD vs. rp-nonND**			
	**AUC** **(95% CI)**	**Sens. (%)**	**Spec. (%)**	**Cutoff (pg/mL)**	**AUC** **(95% CI)**	**Sens. (%)**	**Spec. (%)**	**Cutoff (pg/mL)**
CSF t-tau	0.948 (0.905–0.992)	86.2	97.8	1757	0.889 (0.837–0.941)	86.2	79.1	1739
CSF 14-3-3	0.942 (0.909–0.975)	86.8	88.8	20,700	0.813 (0.736–0.890)	93.7	60.4	16,500
CSF NfL	0.803 (0.714–0.891)	90.8	67.3	2093	0.528 (0.416–0.641)	87.7	29.1	22,000
pl-GFAP	0.762 (0.688–0.837)	64.3	78.2	633	0.758 (0.673–0.843)	80.3	64.5	439
pl-tau	0.836 (0.773–0.898)	74.5	76.7	4	0.775 (0.702–0.848)	45.7	95.5	10
pl-NfL	0.709 (0.609–0.809)	87.1	53.3	49	0.488 (0.377–0.599)	90.1	27.6	340

^1^ Atypical sCJD includes MV2K, MM(V)2C, MM2T, and VV1 sCJD subtypes. Abbreviations: AUC, area under the curve; CI, confidence interval; CSF, cerebrospinal fluid; GFAP, glial fibrillary acidic protein; NfL, neurofilament light chain; np-RPD, non-prion rapidly progressive dementia; rp-ND, neurodegenerative np-RPD; pl-, plasma; t-tau, total tau.

**Table 4:** Associations of plasma and GFAP levels with survival time in the whole sCJD cohort and after stratification according to the clinicopathological subtype.

	Survival Time	Univariate Cox Regression		Multivariate Cox Regression ^1^	
	Median ± IQR (Months)	HR (95% CI)	*p* Value	HR (95% CI)	*p* Value
**Whole CJD cohort (N = 121)**					
Continuous value	1.7 (0.5–3.9)	1.27 (1.00–1.63)	0.050	1.16 (0.86–1.57)	0.301
Low tertile	2.0 (1.0–4.5)	Ref	Ref	Ref	Ref
Mid tertile	1.8 (1.0–4.0)	0.90 (0.57–1.42)	0.660	0.74 (0.47–1.19)	0.225
High tertile	1.3 (0.5–2.7)	1.36 (0.88–2.12)	0.164	1.21 (0.74–1.98)	0.427
**MM(V)1 + VV2 sCJD (N = 92)**					
Continuous value	1.1 (0.5–2.0)	0.98 (0.68–1.40)	0.925	0.89 (0.59–1.36)	0.618
Low tertile	1.0 (0.6–2.0)	Ref	Ref	Ref	Ref
Mid tertile	1.5 (0.9–3.0)	0.63 (0.36–1.09)	0.102	0.57 (0.33–1.00)	0.053
High tertile	1.0 (0.5–2.0)	0.96 (0.57–1.60)	0.882	0.93 (0.52–1.64)	0.809
**Atypical sCJD ^2^ (N = 29)**					
Continuous value	8.0 (4.9–12.0)	1.01 (0.62–1.64)	0.962	1.07 (0.57–2.03)	0.817
Low tertile	9.4 (6.0–11.0)	Ref	Ref	Ref	Ref
Mid tertile	7.0 (3.5–16.0)	0.40 (0.14–1.17)	0.097	0.39 (0.12–1.30)	0.128
High tertile	7.0 (3.0–11.0)	0.89 (0.34–2.34)	0.822	0.95 (0.27–3.32)	0.939

^1^ All multivariate Cox regression analyses included the codon 129 genotype, age at sampling, and time from onset to sample collection as covariates. ^2^ Atypical sCJD includes the MV2K, MM(V)2C, MM2T, and VV1 sCJD subtypes. Bold values indicate statistically significant hazard ratios. Abbreviations: CI, confidence interval; CSF, cerebrospinal fluid; GFAP, glial fibrillary acidic protein; HR, hazard ratio; IQR, interquartile range; Ref, reference; sCJD, sporadic Creutzfeldt–Jakob disease.

**Table 5:** Diagnostic categories of np-RPD cohort (*n* = 94).

np-RPD	Pathological (*n* = 15)	Clinical (*n* = 79)
**rp-nonND**	10	38
Inflammatory	5	18
Toxic-Metabolic	1	10
Neoplastic	1	2
Vascular	3	8
**rp-ND**	5	41
AD	4	25
AD + LBD	1	10
DLB	-	3
FTD	-	3

Abbreviations: AD, Alzheimer’s disease; LBD, Lewy body disease; FTD, frontotemporal dementia; np-RPD, non-prion rapidly progressive dementia; rp-ND, neurodegenerative np-RPD.

## Data Availability

The data presented in this study are available upon reasonable request from the corresponding author.

## Supplementary Materials

The following supporting information can be downloaded at: https://www.mdpi.com/article/10.3390/ijms25105106/s1.
