# Supplementary material for: Diagnostic and Prognostic Value of Plasma GFAP in Sporadic Creutzfeldt–Jakob Disease in the Clinical Setting of Rapidly Progressive Dementia
Source: Int J Mol Sci. 2024 May 8;25(10):5106. doi: 10.3390/ijms25105106 (PMC11121136; doi:10.3390/ijms25105106)
Supplement: Supplementary file 1 [file ijms-25-05106-s001.zip › ijms-2986403-supplementary.pdf]

Diagnostic and Prognostic Value of Plasma GFAP in Sporadic Creutzfeldt–Jakob Disease in the Clinical Setting of Rapidly Progressive Dementia

Giuseppe Mario Bentivenga [1], Simone Baiardi [1,2], Andrea Mastrangelo [1], Corrado Zenesini [2], Angela Mammana [2], Marcello Rossi [2], Barbara Polischi [2], Sabina Capellari [1,2] and Piero Parchi [1,2,*]

[1] Department of Biomedical and Neuromotor Sciences (DiBiNeM), University of Bologna, 40139, Bologna, Italy; giuseppe.bentivenga@studio.unibo.it (G.M.B.); simone.baiardi6@unibo.it (S.B.); andrea.mastrangelo4@studio.unibo.it; sabina.capellari@unibo.it (S.C.)
[2] IRCCS Istituto delle Scienze Neurologiche di Bologna, 40139, Bologna, Italy; corrado.zenesini@isnb.it (C.Z.); angela.mammana2@unibo.it; marcello.rossi@ausl.bologna.it (M.R.); barbara.polischi@isnb.it (B.P.)
* Correspondence: piero.parchi@unibo.it (PP); Tel.: +39-051-4966740

SUPPLEMENTARY MATERIAL

Table S1. Distribution of CSF and plasma surrogate neurodegeneration biomarkers levels in the subgroups of the sCJD cohort

**Diagnostic group**	**N**	**CSF t-tau (pg/mL)**	**CSF 14-3-3 (AU/mL)**	**CSF NfL (pg/mL)**	**pl-NfL (pg/mL)**	**pl-tau (pg/mL)**
MM(V)1	63	6768 (3315-11500)	74650 (38200-135000)	6053 (3350-9127)	112 (66-204)	19 (10-38)
VV2	35	11575 (7961-14680)	121000 (78200-178000)	14600 (10800-29500)	142 (105-249)	5 (3-6)
MV2K	26	1867 (1242-2595)	22100 (16600-29300)	7442 (3210-9657)	88 (50-164)	6 (3-15)
MM(V)2C	5	1185 (1134-1578)	23700 (16600-27000)	4187 (3590-4563)	58 (52-77)	7 (4-12)
MM2T	1	352	3699	2877	49	4
VV1	2	3620, 3325	53900, 68200	45700, 11100	275, 34	2, -

Both patients with a definite diagnosis of a specific subtype and patients with a probable diagnosis and a high level of certainty for a given subtype are included.
Biomarker data are presented as median (IQR)
Abbreviations: Aβ, amyloid beta; CSF, cerebrospinal fluid; NfL, neurofilament light chain; p-tau, phospho-tau181; t-tau, total tau

Table S2. Associations of CSF and plasma biomarkers with survival time in the sCJD cohort

Biomarker		Survival time	Univariate Cox regression		Multivariate Cox regression	
		Median ± IQR (months)	HR (95% CI)	P value	HR (95% CI)	P value
**CSF t-tau (N = 120)**	Continuous value	1.6 (0.9-3.2)	**1.84 (1.48-2.29)**	**<0.001**	**1.60 (1.25-2.06)**	**<0.001**
	Low tertile	3.7 (1.2-9.4)	Ref	Ref	Ref	Ref
	Mid tertile	1.6 (1.0-3.0)	**2.06 (1.29-3.12)**	**0.002**	1.37 (0.77-2.42)	0.272
	High tertile	0.8 (0.5-2.0)	**3.70 (2.24- 6.10)**	**<0.001**	**2.97 (1.56-5.63)**	**<0.001**
**CSF 14-3-3 (N = 118)**	Continuous value	1.6 (0.9-3.5)	**1.89 (1.51-2.37)**	**<0.001**	**1.78 (1.38-2.31)**	**<0.001**
	Low tertile	3.7 (1.1-9.4)	Ref	Ref	Ref	Ref
	Mid tertile	1.8 (1.0-3.0)	**2.08 (1.29-3.33)**	**0.002**	**1.85 (1.06-3.21)**	**0.028**
	High tertile	0.8 (0.5-2.0)	**3.57 (2.17-5.87)**	**<0.001**	**3.35 (1.81-6.19)**	**<0.001**
**CSF NfL (N = 120)**	Continuous value	1.6 (0.9-3.2)	**1.25 (1.03-1.53)**	**0.023**	**1.39 (1.08-1.80)**	**0.010**
	Low tertile	2.0 (1.0-6.0)	Ref	Ref	Ref	Ref
	Mid tertile	1.3 (0.8-3.0)	1.46 (0.94-2.28)	0.091	1.45 (0.91-2.32)	.115
	High tertile	1.8 (0.5-3.0)	1.51 (0.95-2.38)	0.076	**2.14 (1.23-3.72)**	**0.007**
**CSF GFAP (N = 60)**	Continuous value	1.6 (1.0-3.4)	1.04 (0.73-1.49)	0.791	0.93 (0.64-1.35)	0.722
	Low tertile	1.8 (0.6-6.6)	Ref	Ref	Ref	Ref
	Mid tertile	1.6 (1.0-3.0)	1.39 (0.72-2.66)	0.319	1.39 (0.68-2.82)	0.357
	High tertile	1.7 (1.0-3.9)	1.42 (0.73-2.75)	0.295	1.07 (0.50-2.27)	0.853
**pl-NfL (N = 121)**	Continuous value	1.7 (0.5-3.9)	**1.25 (1.01-1.54)**	**0.036**	1.23 (0.98-1.54)	0.068
	Low tertile	2.1 (1.0-6.0)	Ref	Ref	Ref	Ref
	Mid tertile	1.6 (0.8-3.1)	1.37 (0.87-2.14)	0.166	1.29 (0.81-2.06)	0.271
	High tertile	1.4 (0.5-3.0)	1.56 (0.99-2.44)	0.053	**1.61 (1.00-2.59)**	**0.049**
**pl-tau (N = 110)**	Continuous value	1.8 (0.9-3.6)	**1.28 (1.08-1.52)**	**0.004**	**1.28 (1.04-1.58)**	**0.016**
	Low tertile	2.4 (1.0-6.0)	Ref	Ref	Ref	Ref
	Mid tertile	1.6 (1.0-3.2)	1.39 (0.87-2.22)	0.164	**1.80 (1.06-3.05)**	**0.028**
	High tertile	1.1 (0.5-2.3)	**1.84 (1.16-2.92)**	**0.009**	**1.89 (1.04-3.42)**	**0.034**

All multivariate Cox regression analyses included codon 129 genotype, age at sampling and time from onset to sample collection as covariates.

Bold values indicate statistically significant hazard ratios.

Abbreviations: CI, confidence interval; CSF, cerebrospinal fluid; GFAP, Glial Fibrillary Acidic Protein; HR, hazard ratio; IQR, interquartile range; Ref, reference; sCJD, sporadic Creutzfeldt-Jakob disease

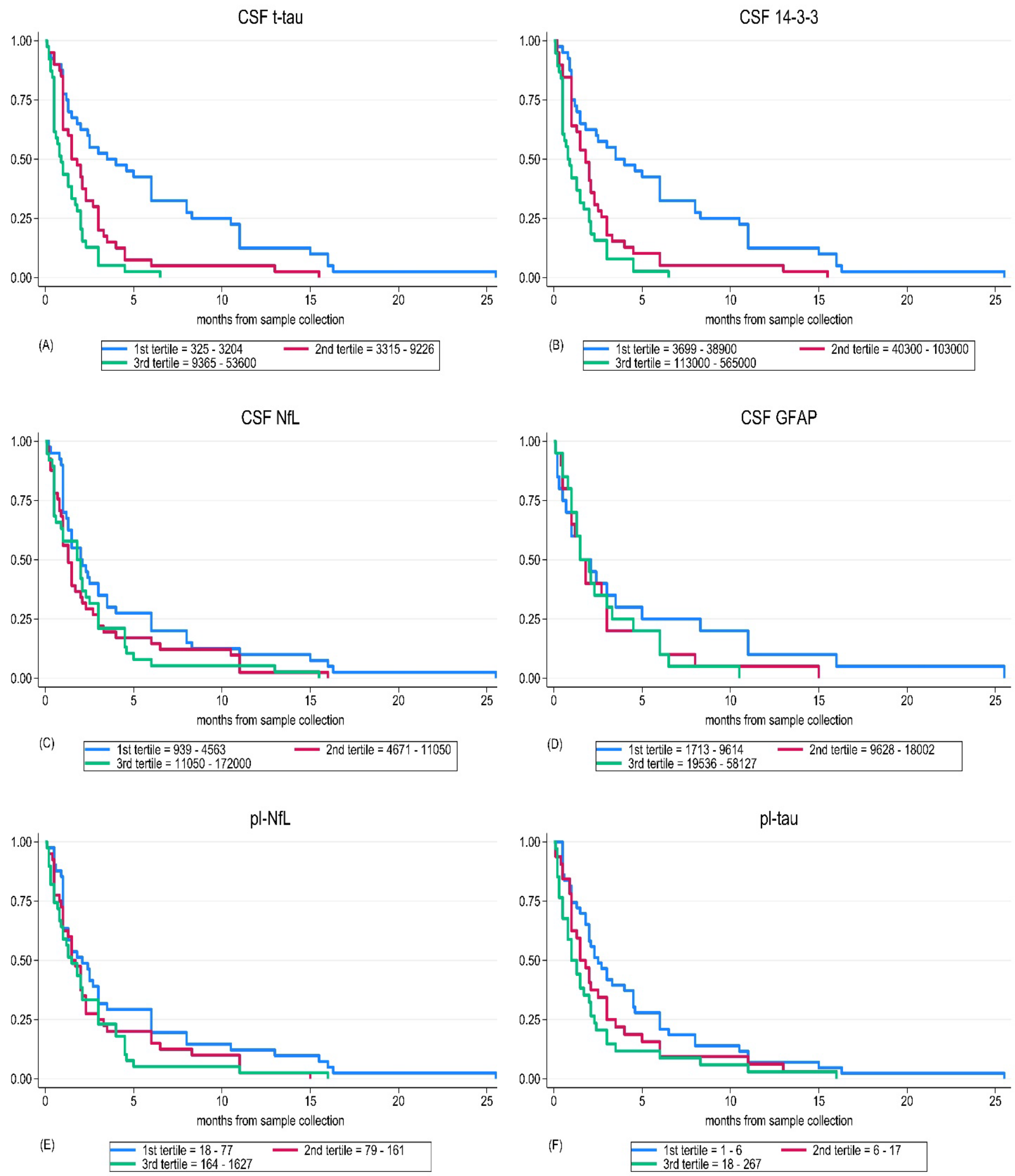

Figure S1. Prognostic value of CSF t-tau (A), 14-3-3 (B), CFS NfL (C), CSF GFAP (D), pl- NfL (E), pl-tau (F). Survival curves in patients of the whole sCJD cohort according to the values of CSF and plasma biomarkers. CSF, cerebrospinal fluid; GFAP, Glial Fibrillary Acidic Protein; NfL, neurofilament light chain; t-tau, total tau

Supplementary methods

Probable sCJD patients' classification

The classification of probable sCJD subtypes was determined by the consensus of three evaluators (GMB, SB, and PP) after reviewing genetic testing results (codon 129 genotype), clinical features, CSF biomarkers, and brain MRI as previously described [1,2].

Probable sCJD VV2 cases were homozygotes VV at codon 129, showed prominent rapidly progressive early ataxia and a disease duration < 12 months, were positive at prion RT-QuIC [3], and had at least 2 of the following: positive 14-3-3 WB assay, and/or 14-3-3 > 23400 AU/ml, and/or t-tau levels > 1250 pg/ml, prominent striatum and/or thalamic involvement at brain MRI in the early phase of the disease [3–7]. They showed averagely high values of CSF 14-3-3 and t-tau (121000 (78200-178000) AU/ml and 11575 (7961-14680) pg/ml, respectively), as described [3,8]. Patients (129-VV) with clinical features highly suggestive of the VV1 subtype (i.e., age at onset ≤36 years, disease duration ≥17 months, and predominant cortical symptoms/signs without ataxia [7]) were excluded and classified accordingly. Probable sCJD MV2K were heterozygotes MV at codon 129, presented with prominent ataxia and/or cognitive decline at onset, had a disease duration > 8 months [4,6], were positive at prion RT-QuIC [3] and brain MRI (DWI/FLAIR sequences) [5]. Furthermore, probable MV2K patients showed relatively low CSF 14-3-3 and t-tau levels (22100 (16600-29300) and 1867 (1242-2595), respectively) as previously described [3,8,9]. Patients (129-MV) exhibiting clinical features highly suggestive of the MV1 subtype (disease duration < 8 months and presenting with a multisystemic neurological syndrome) were excluded and classified accordingly as previously reported [1,2]. Probable sCJD MM2C were homozygotes MM at codon 129, showed a prominent cognitive decline at onset, had a disease duration > 8 months [4,6], and were positive at prion RT-QuIC [3] and brain MRI (DWI/FLAIR sequences) [5]. Moreover, probable MM2C showed low levels of CSF 14-3-3 and t-tau (23700 (16600-27000) pg/ml and 1185 (1134-1578) pg/ml, respectively) as described [3,8,9]. Patients (129 MM) with disease duration < 6 months and presenting with a multisystemic neurological syndrome were classified as MM1.

Supplementary references

1. Bentivenga, G.M.; Baiardi, S.; Mastrangelo, A.; Zenesini, C.; Mammana, A.; Polischi, B.; Capellari, S.; Parchi, P. Diagnostic and Prognostic Value of Cerebrospinal Fluid SNAP-25 and Neurogranin in Creutzfeldt-Jakob Disease in a Clinical Setting Cohort of Rapidly Progressive Dementias. *Alzheimers Res Ther* **2023**, *15*, 150, doi:10.1186/s13195-023-01300-y.

2. Mastrangelo, A.; Baiardi, S.; Zenesini, C.; Poleggi, A.; Mammana, A.; Polischi, B.; Ladogana, A.; Capellari, S.; Parchi, P. Diagnostic and Prognostic Performance of CSF α-synuclein in Prion Disease in the Context of Rapidly Progressive Dementia. *Alzheimer's Dement.* **2021**, *13*, doi:10.1002/dad2.12214.

3. Lattanzio, F.; Abu-Rumeileh, S.; Franceschini, A.; Kai, H.; Amore, G.; Poggiolini, I.; Rossi, M.; Baiardi, S.; McGuire, L.; Ladogana, A.; et al. Prion-Specific and Surrogate CSF Biomarkers in Creutzfeldt-Jakob Disease: Diagnostic Accuracy in Relation to Molecular Subtypes and Analysis of Neuropathological Correlates of p-Tau and Aβ42 Levels. *Acta Neuropathol* **2017**, *133*, 559–578, doi:10.1007/s00401-017-1683-0.

4. Parchi, P.; Giese, A.; Capellari, S.; Brown, P.; Schulz-Schaeffer, W.; Windl, O.; Zerr, I.; Budka, H.; Kopp, N.; Piccardo, P.; et al. Classification of Sporadic Creutzfeldt-Jakob Disease Based on Molecular and Phenotypic Analysis of 300 Subjects. *Ann Neurol* **1999**, *46(2)*, 224-233.

5. Hermann, P.; Appleby, B.; Brandel, J.-P.; Caughey, B.; Collins, S.; Geschwind, M.D.; Green, A.; Haïk, S.; Kovacs, G.G.; Ladogana, A.; et al. Biomarkers and Diagnostic Guidelines for Sporadic Creutzfeldt-Jakob Disease. *The Lancet Neurol* **2021**, *20*, 235–246, doi:10.1016/S1474-4422(20)30477-4.

6. Parchi, P.; de Boni, L.; Saverioni, D.; Cohen, M.L.; Ferrer, I.; Gambetti, P.; Gelpi, E.; Giaccone, G.; Hauw, J.-J.; Höftberger, R.; et al. Consensus Classification of Human Prion Disease Histotypes Allows Reliable Identification of Molecular Subtypes: An Inter-Rater Study among Surveillance Centres in Europe and USA. *Acta Neuropathol* **2012**, *124*, 517–529, doi:10.1007/s00401-012-1002-8.

7. Baiardi, S.; Magherini, A.; Capellari, S.; Redaelli, V.; Ladogana, A.; Rossi, M.; Tagliavini, F.; Pocchiari, M.; Giaccone, G.; Parchi, P. Towards an Early Clinical Diagnosis of Sporadic CJD VV2 (Ataxic Type). *J Neurol Neurosurg Psychiatry* **2017**, *88*, 764–772, doi:10.1136/jnnp-2017-315942.

8. Abu-Rumeileh, S.; Capellari, S.; Stanzani-Maserati, M.; Polischi, B.; Martinelli, P.; Caroppo, P.; Ladogana, A.; Parchi, P. The CSF Neurofilament Light Signature in Rapidly Progressive Neurodegenerative Dementias. *Alzheimers Res Ther* **2018**, *10*, 3, doi:10.1186/s13195-017-0331-1.

9. Abu-Rumeileh, S.; Baiardi, S.; Polischi, B.; Mammana, A.; Franceschini, A.; Green, A.; Capellari, S.; Parchi, P. Diagnostic Value of Surrogate CSF Biomarkers for Creutzfeldt–Jakob Disease in the Era of RT-QuIC. *J Neurol* **2019**, *266*, 3136–3143, doi:10.1007/s00415-019-09537-0.
